# Supplementary figures and images for: Tumor microenvironment related novel signature predict lung adenocarcinoma survival
Source: PeerJ. 2021 Jan 14;9:e10628. doi: 10.7717/peerj.10628 (PMC7811787; doi:10.7717/peerj.10628)

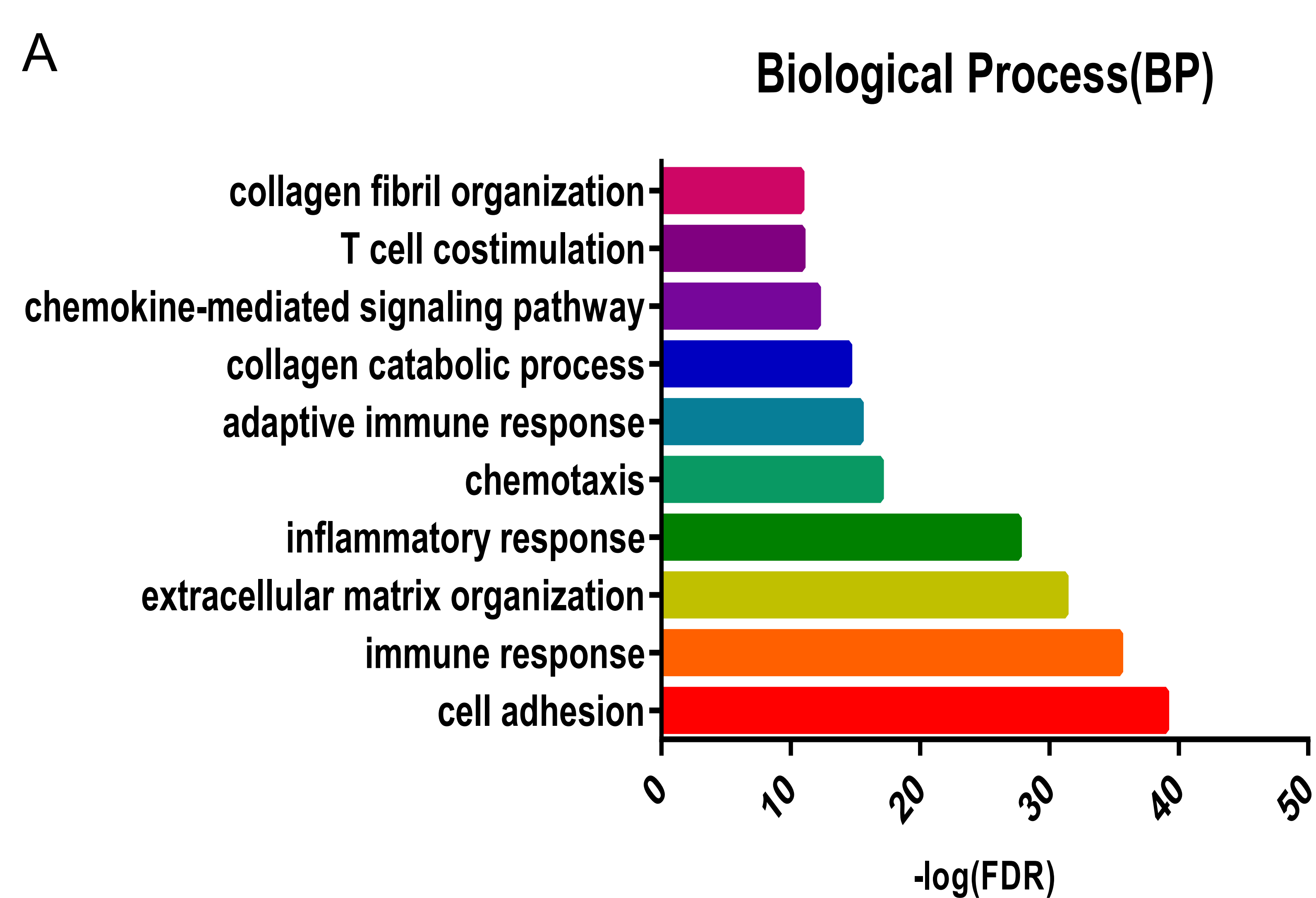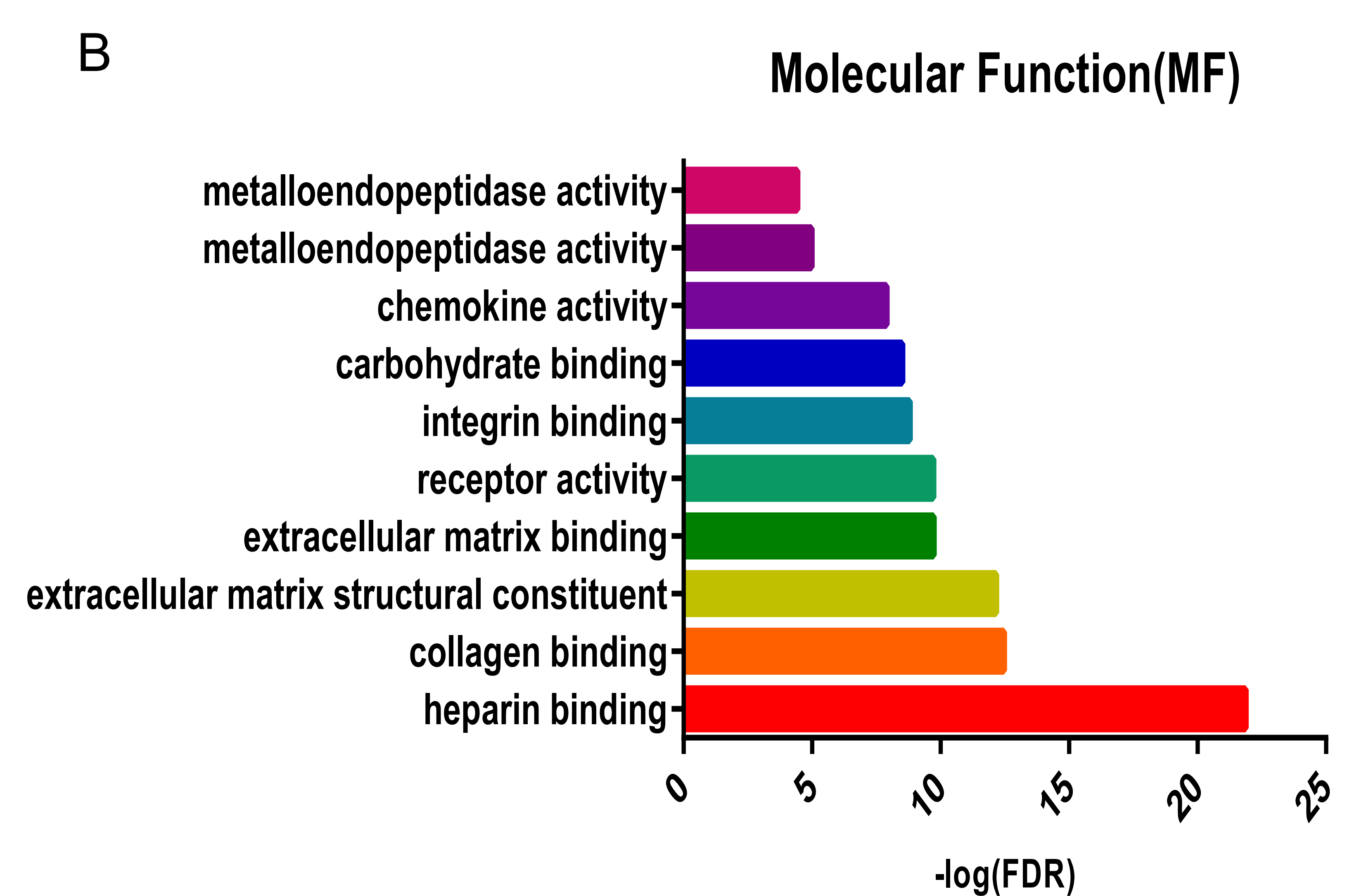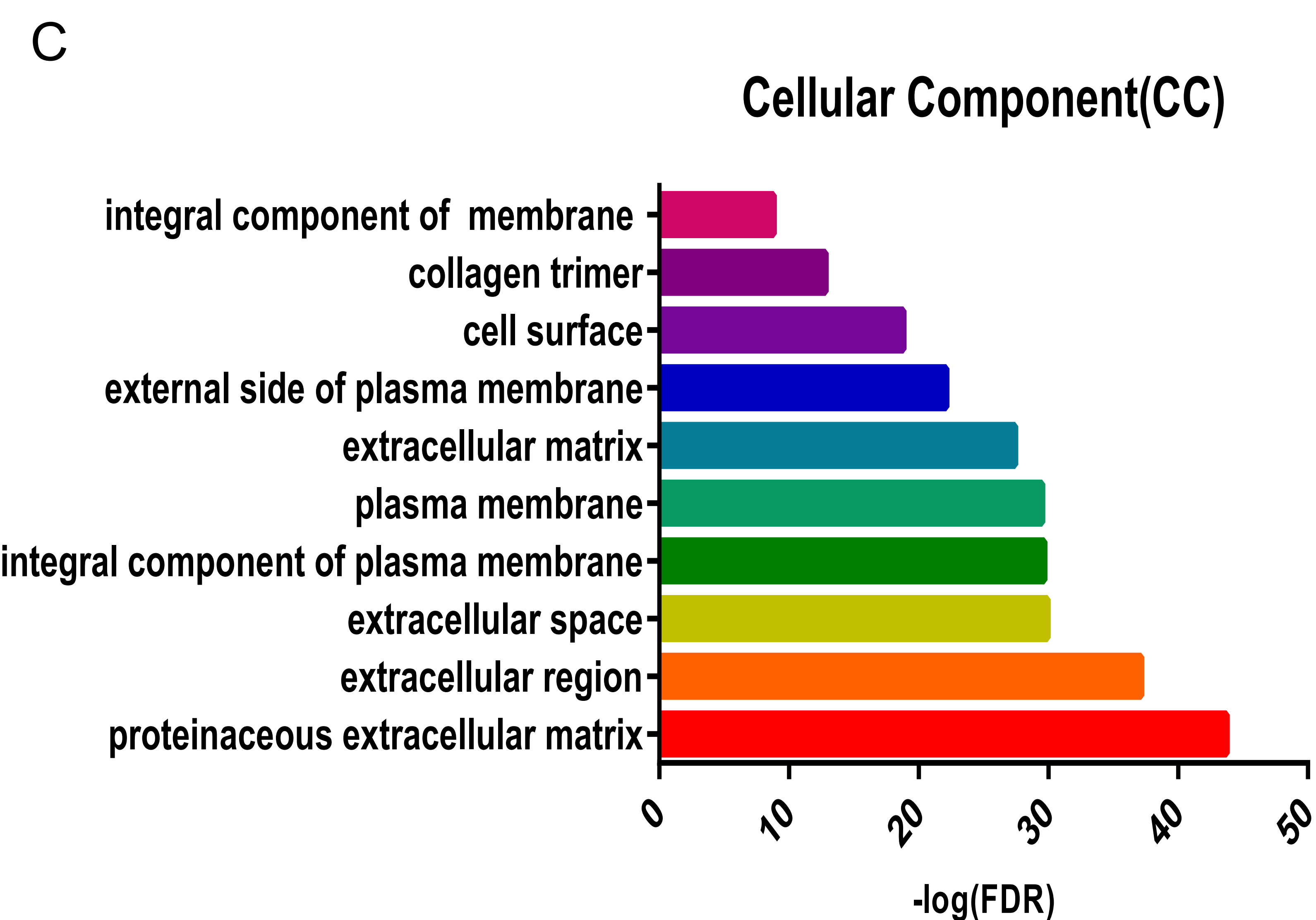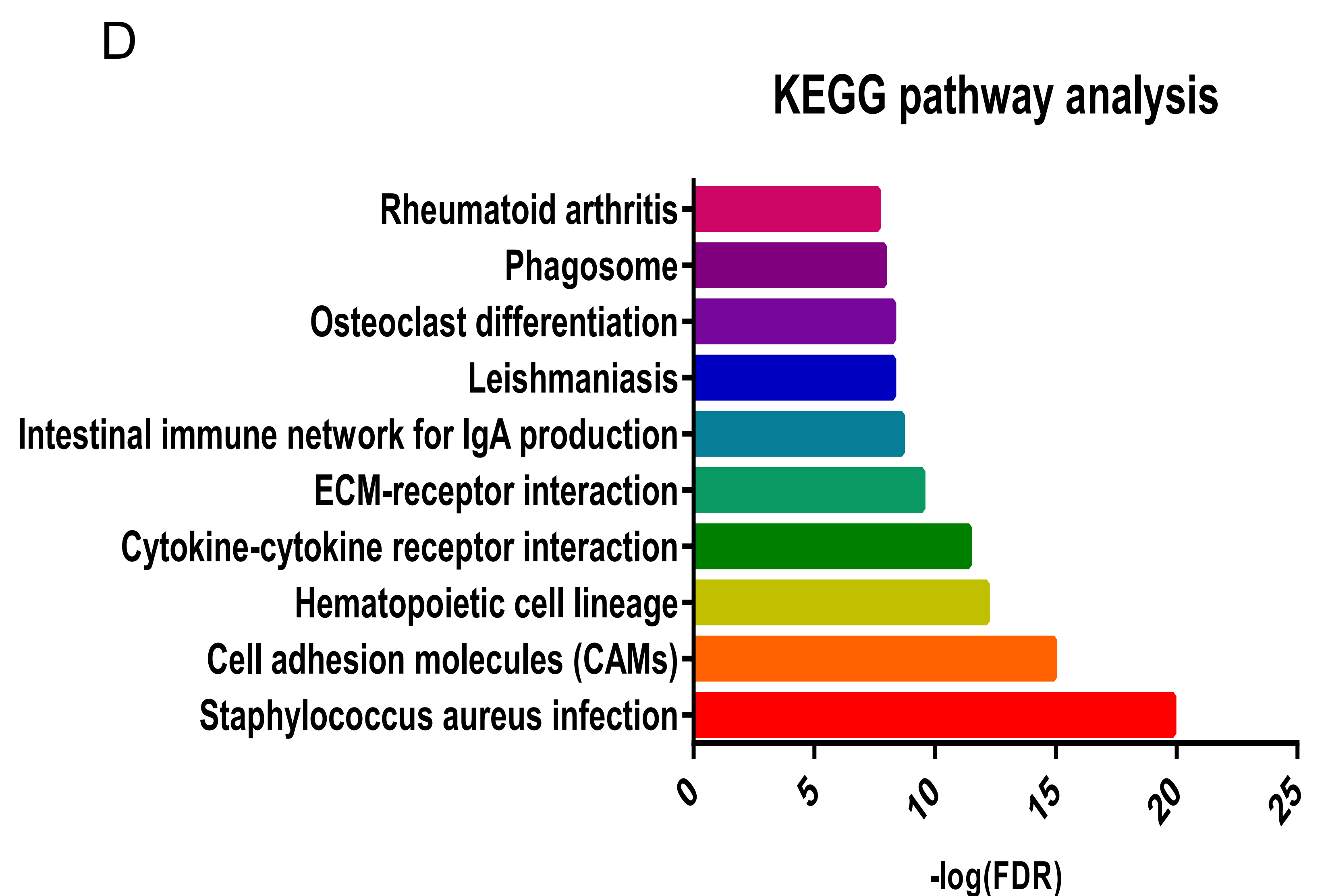

Supplement: Supplemental Information 1 — Top 10 pathways. False discovery rate (FDR) of GO analysis was acquired from DAVID functional annotation tool. p <0.05. (A) biological process, (B) cellular component, (C) molecular function, (D) KEGG pathway. [file peerj-09-10628-s001.pdf]

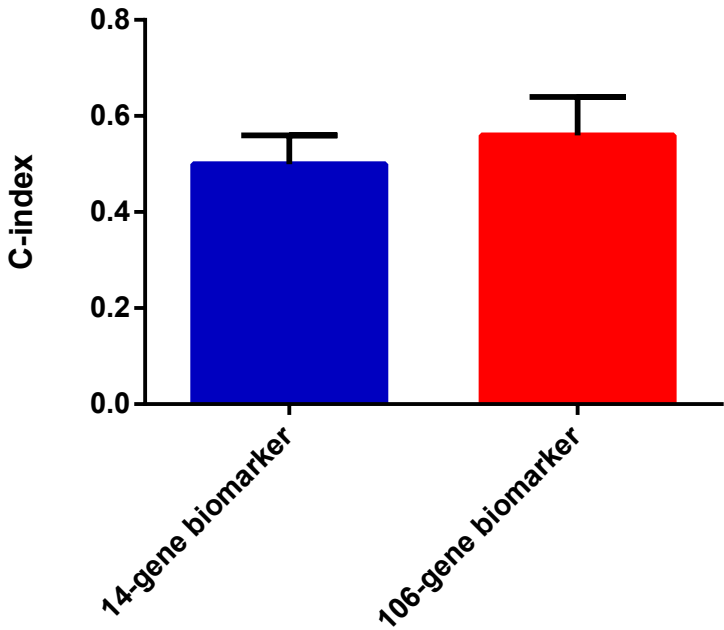

Supplement: Supplemental Information 2 — Comparison of C-index between 14-gene biomarker and 106-gene biomarker in GSE14814 data set. [file peerj-09-10628-s002.pdf]

A

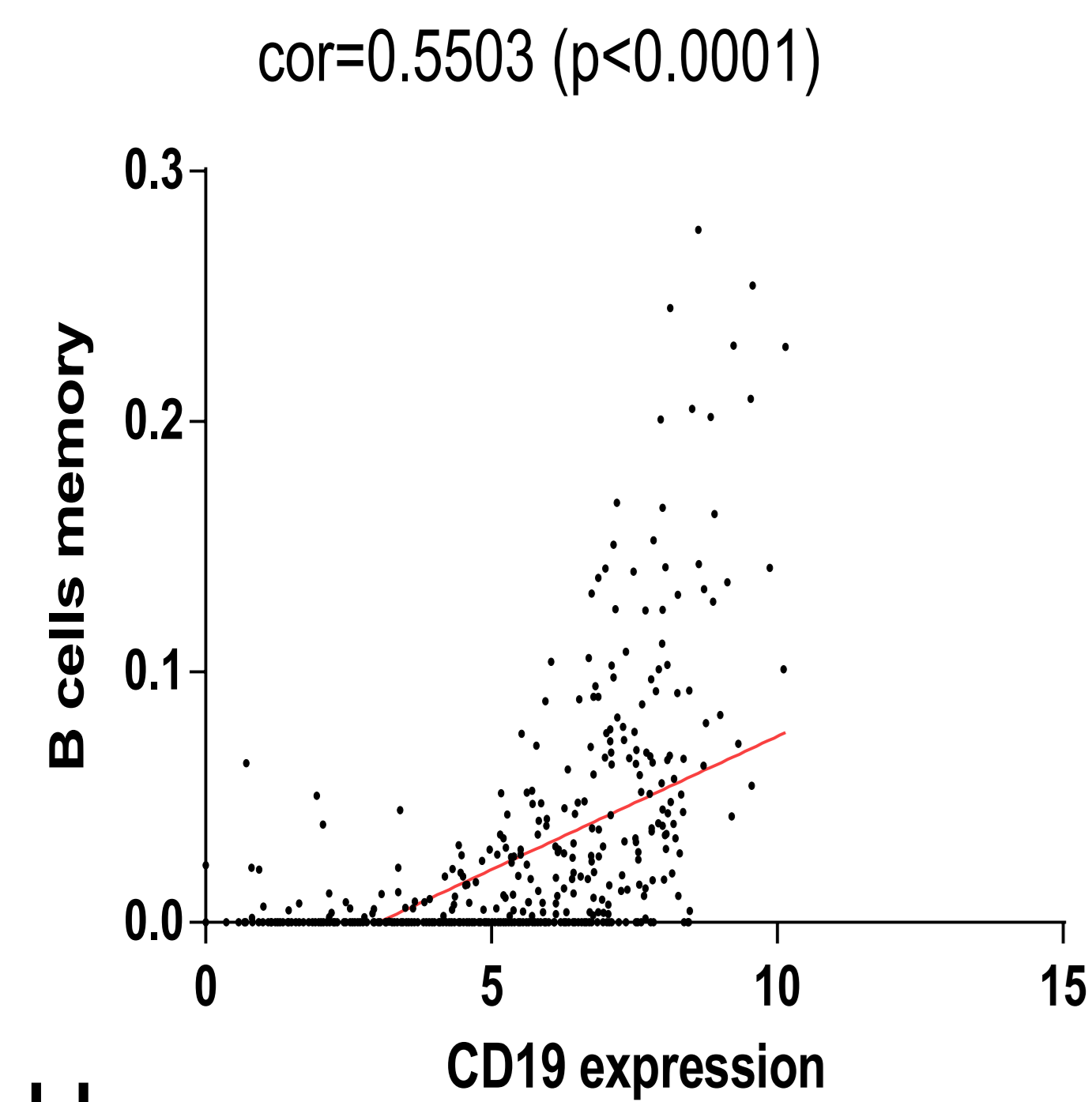

B

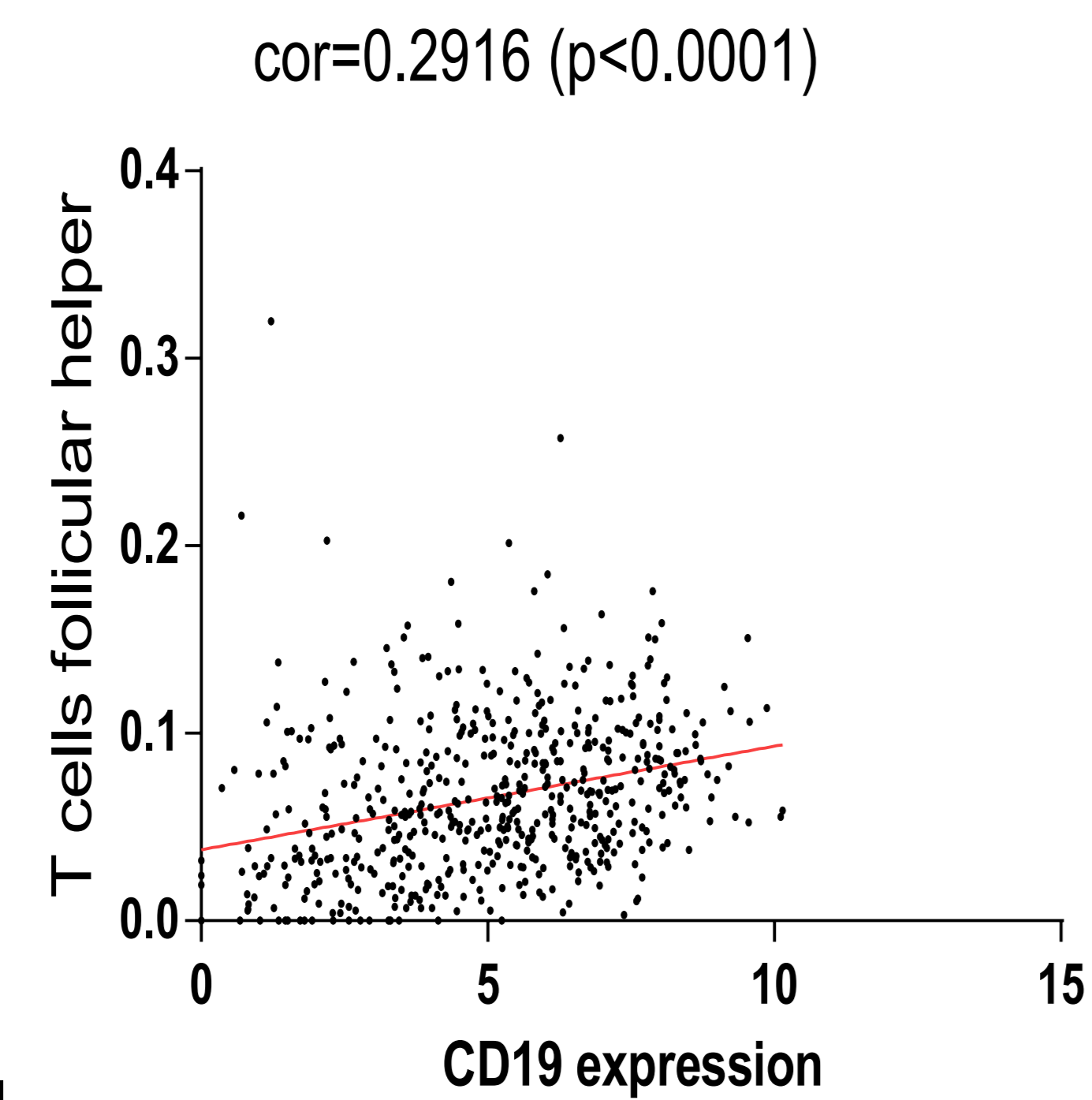

C

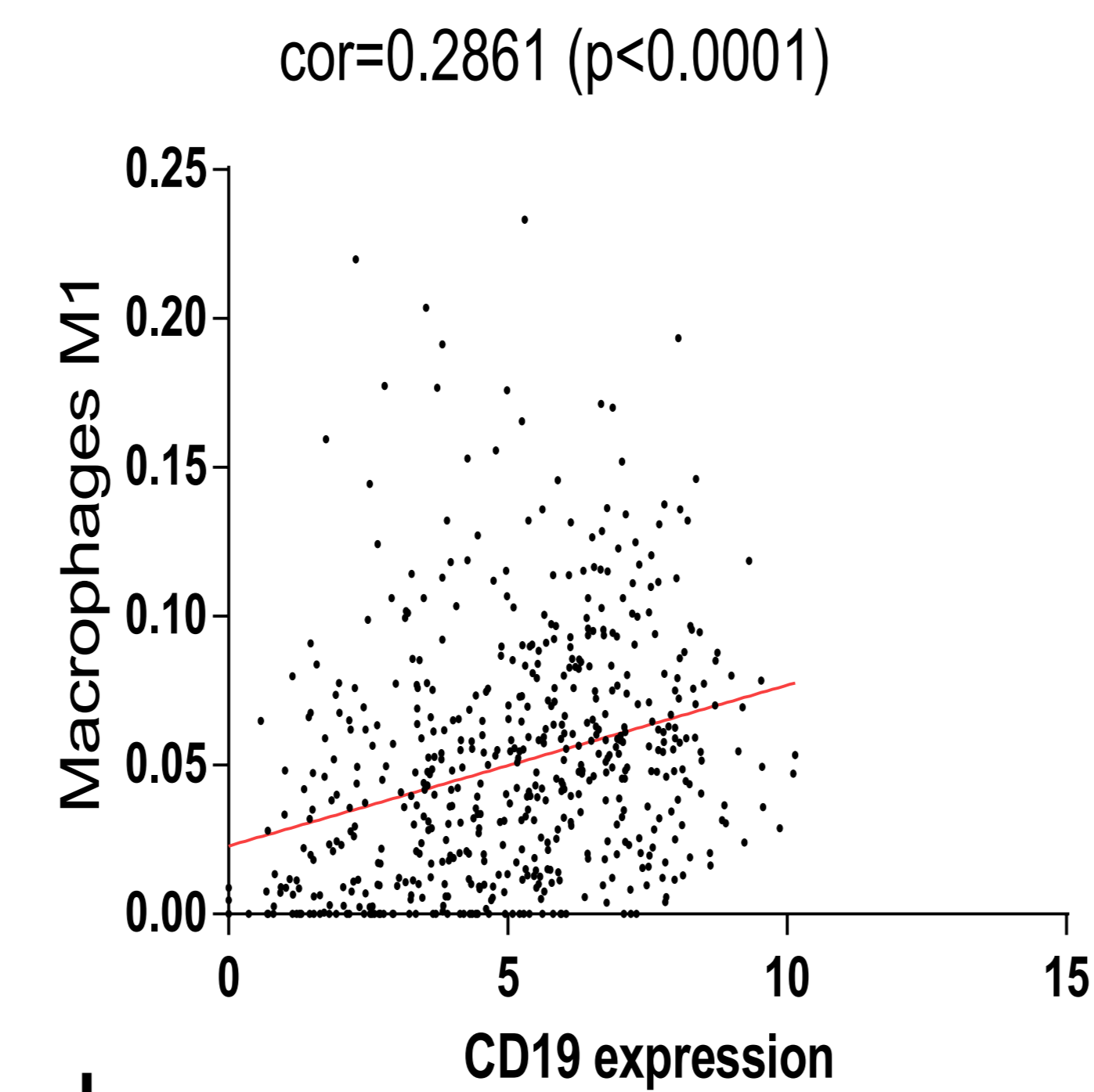

D

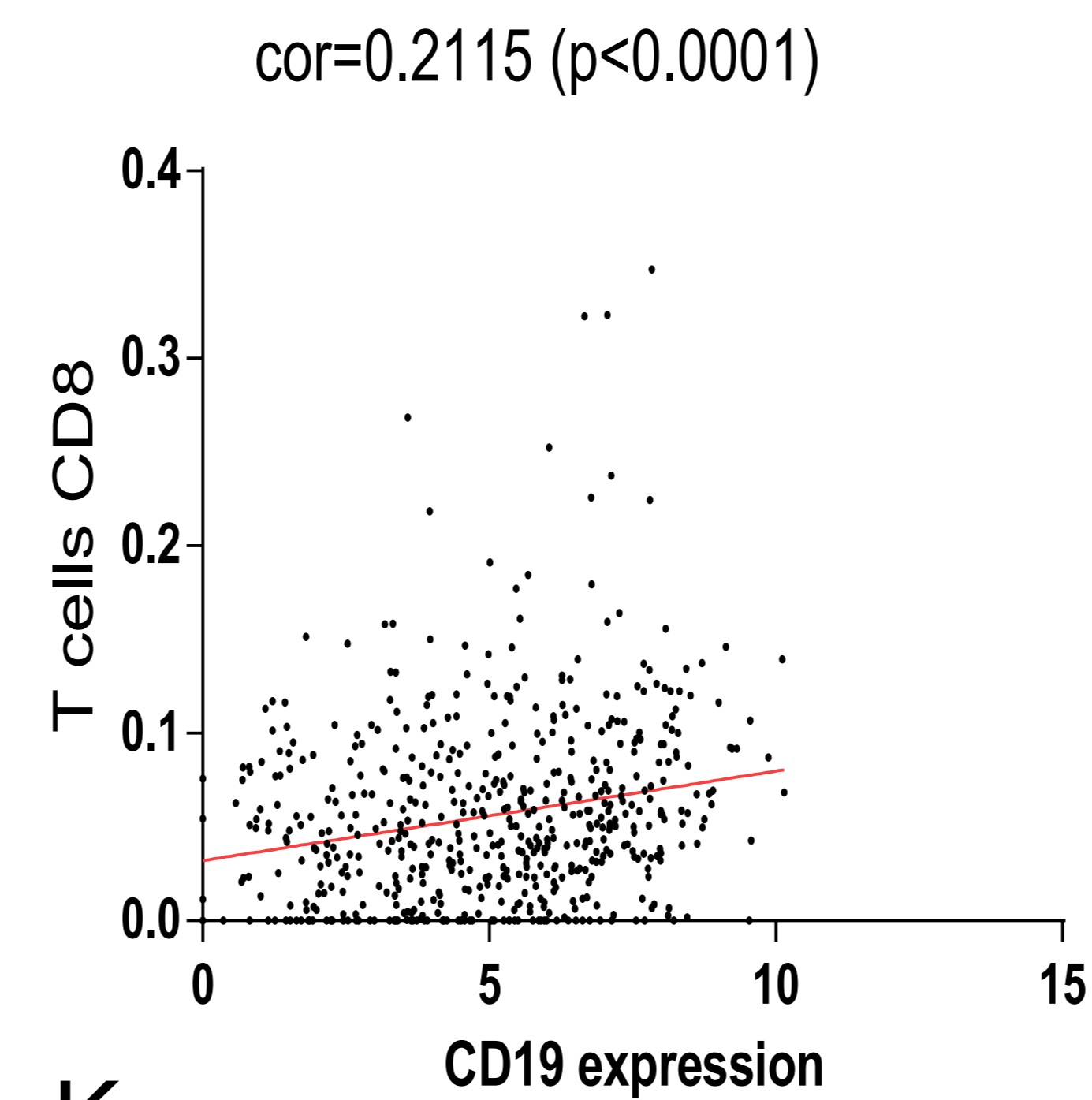

E

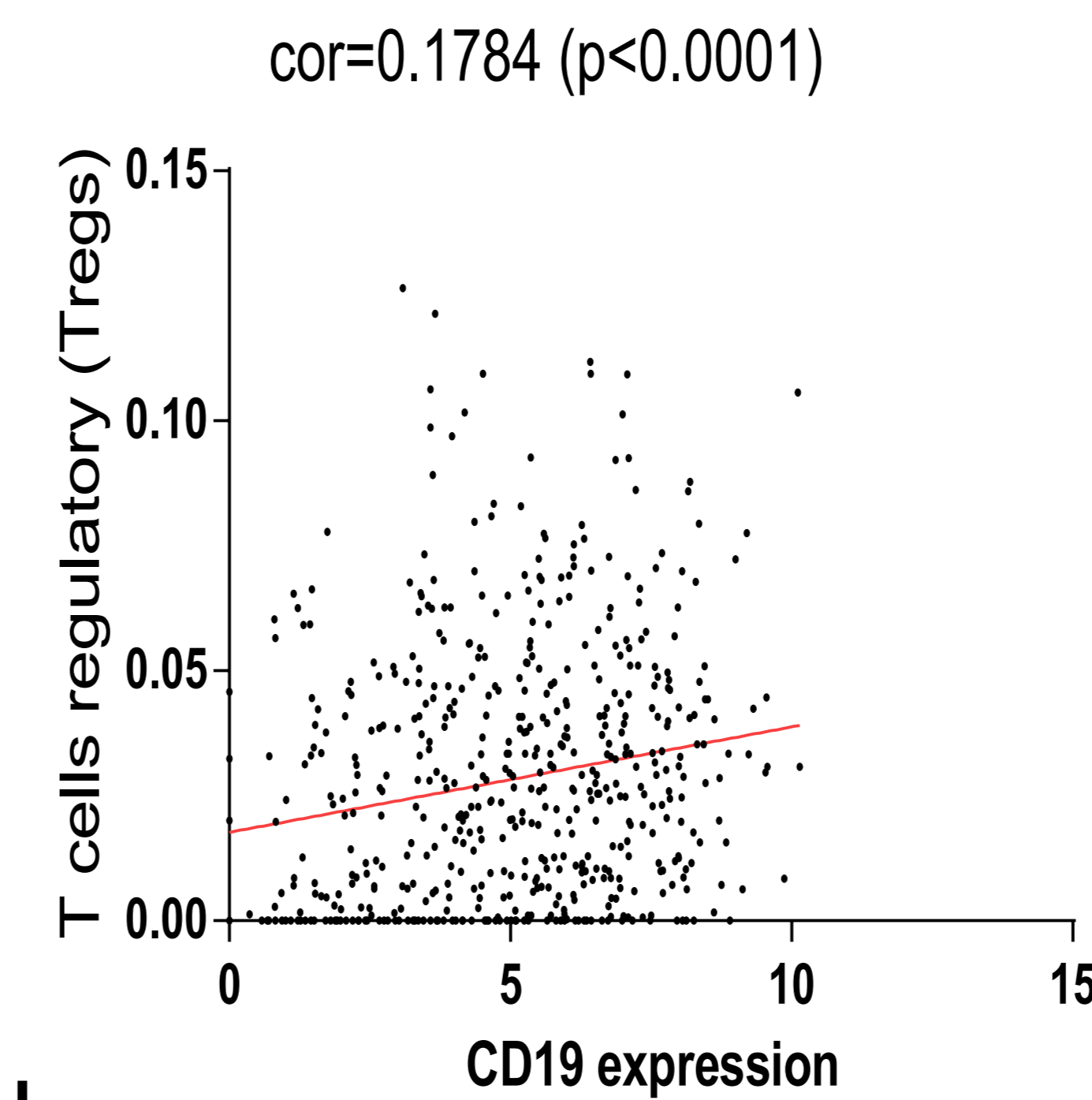

F

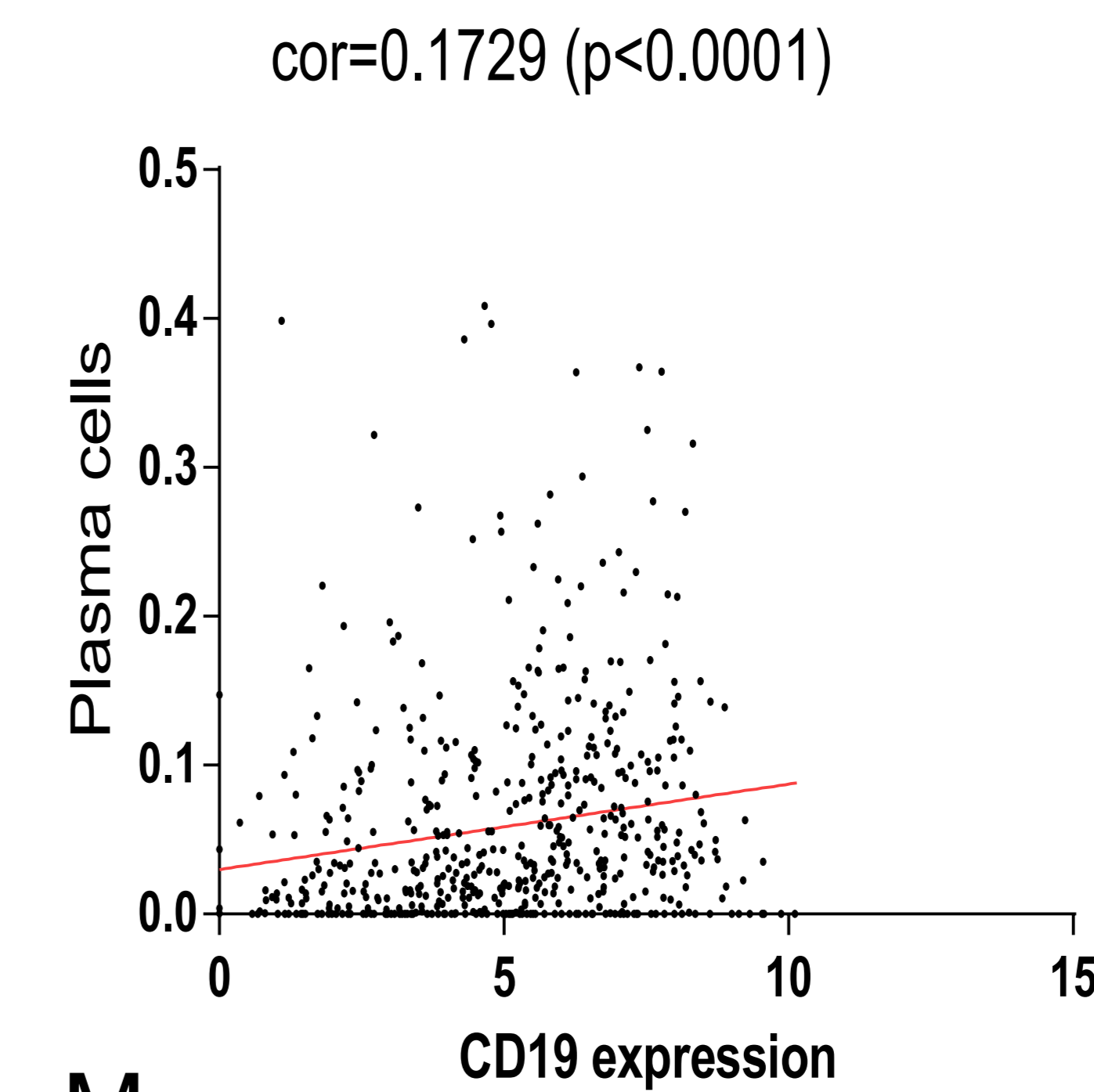

G

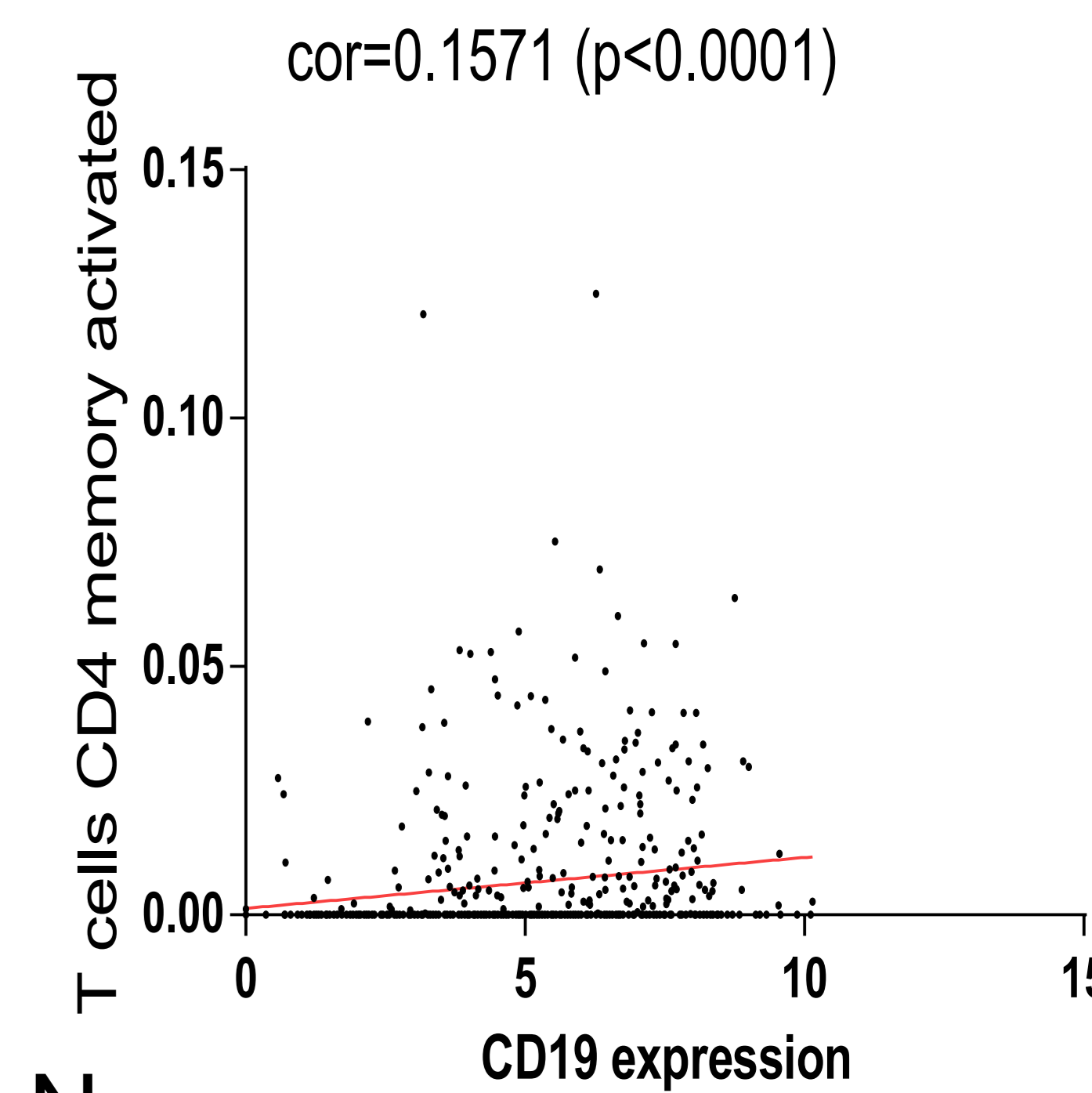

H

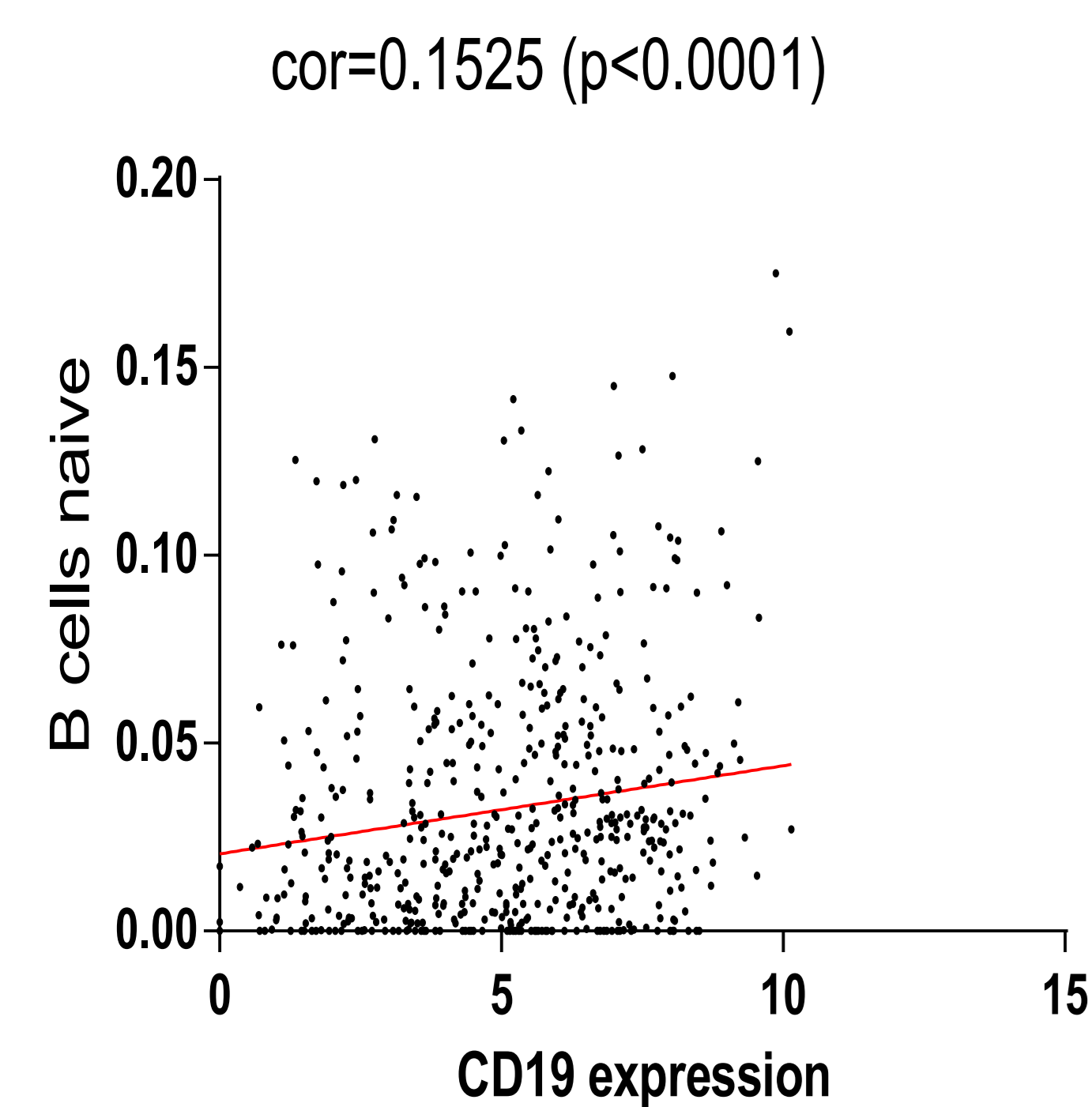

I

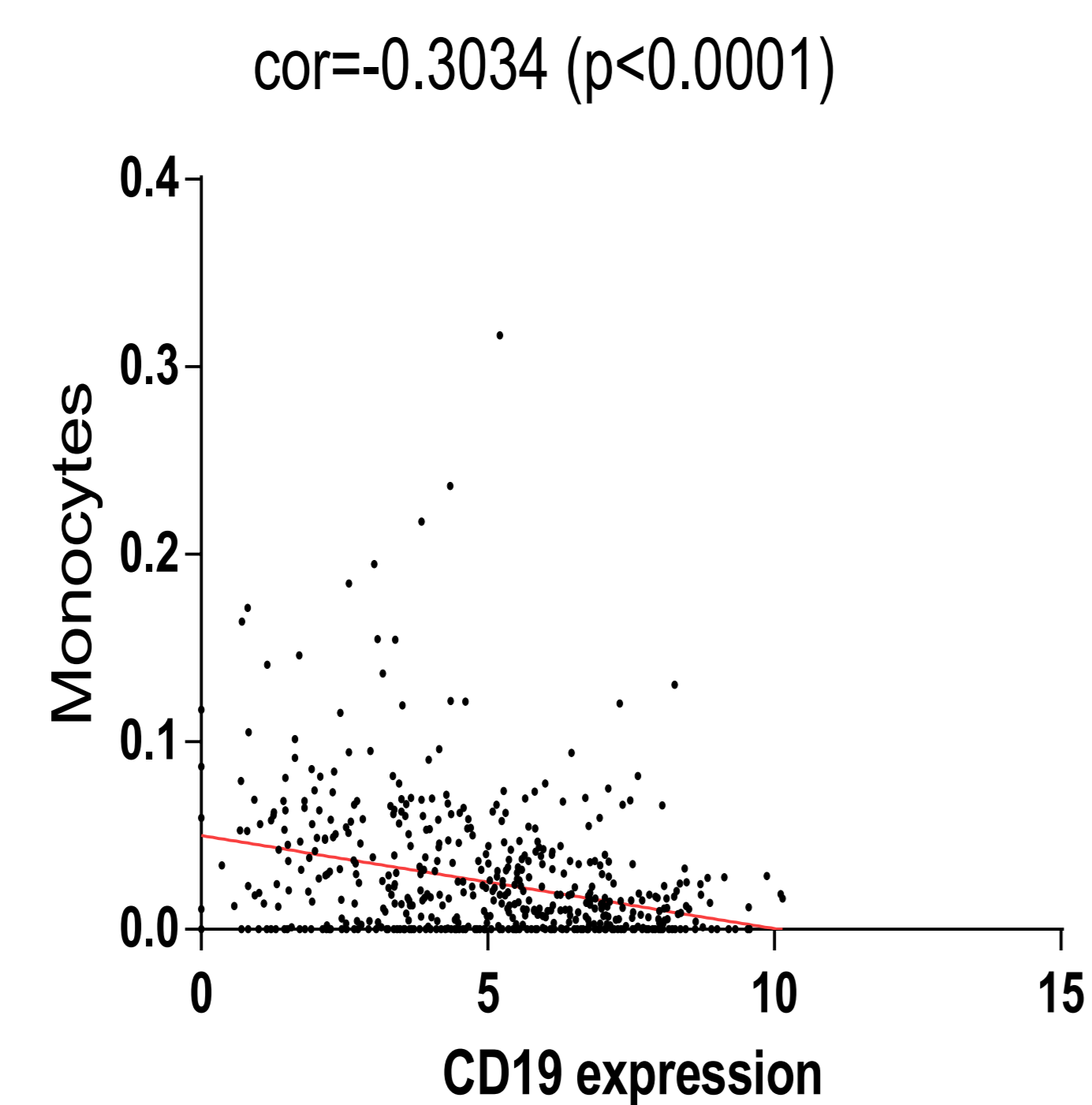

J

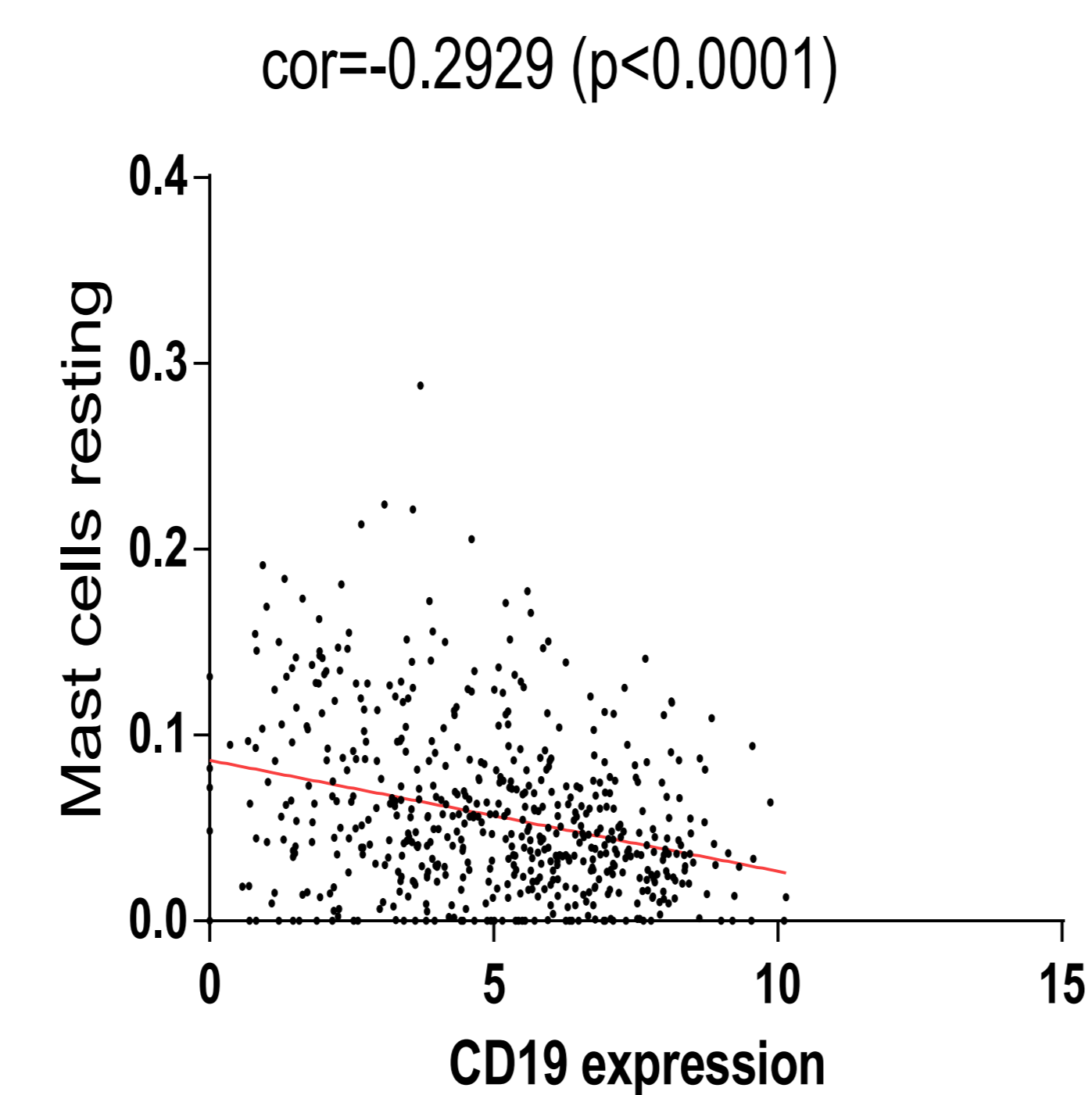

K

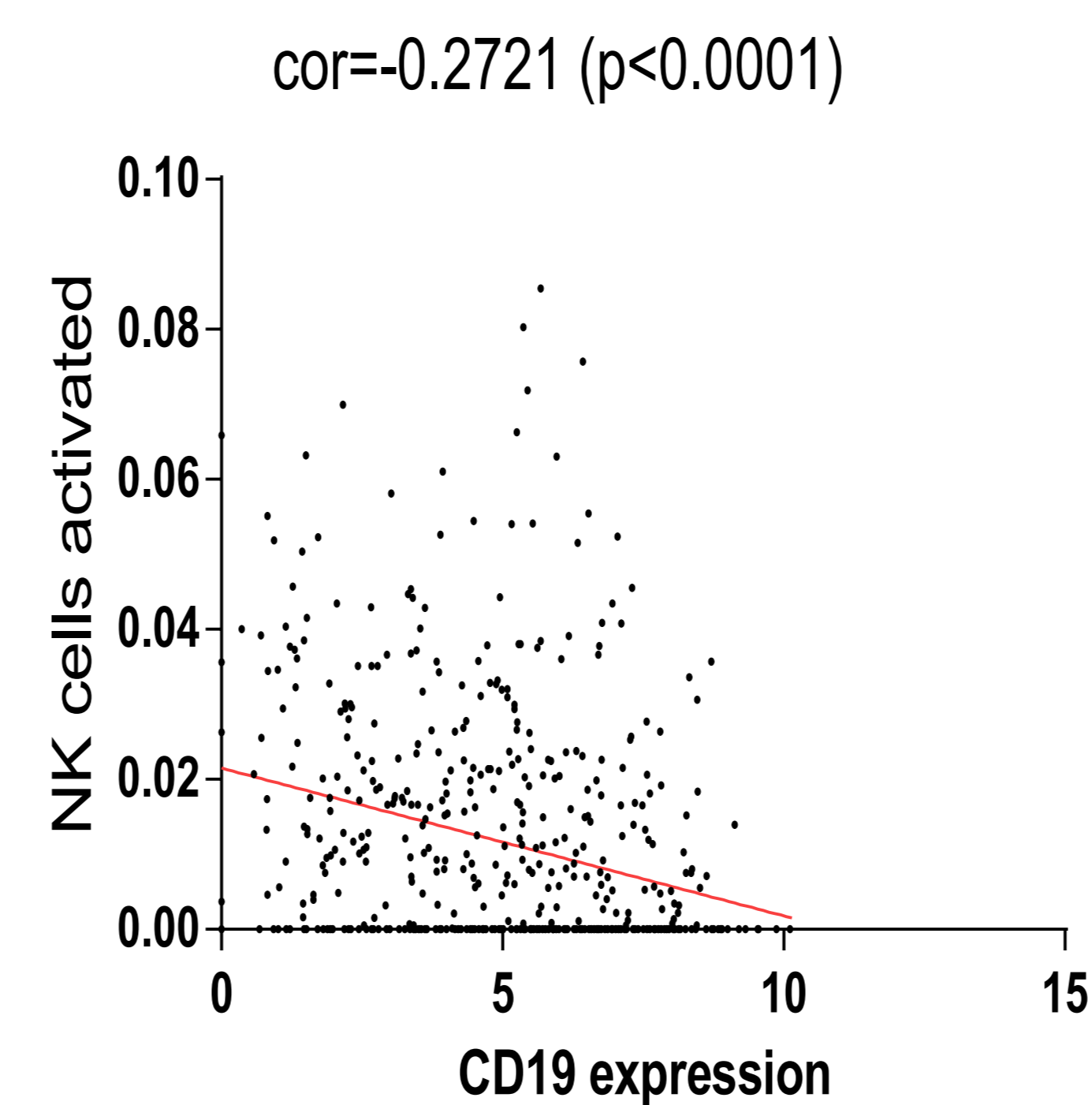

L

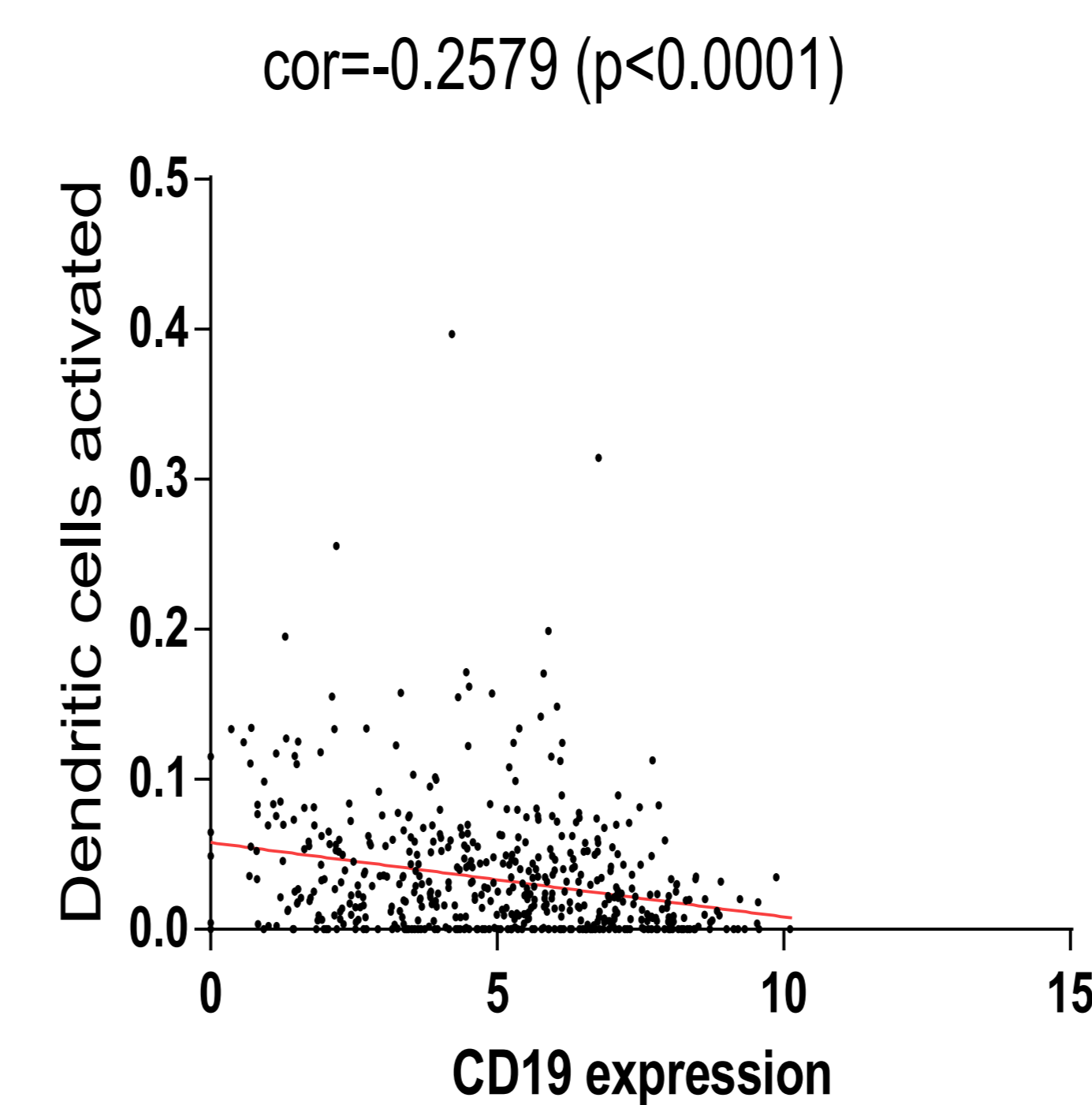

M

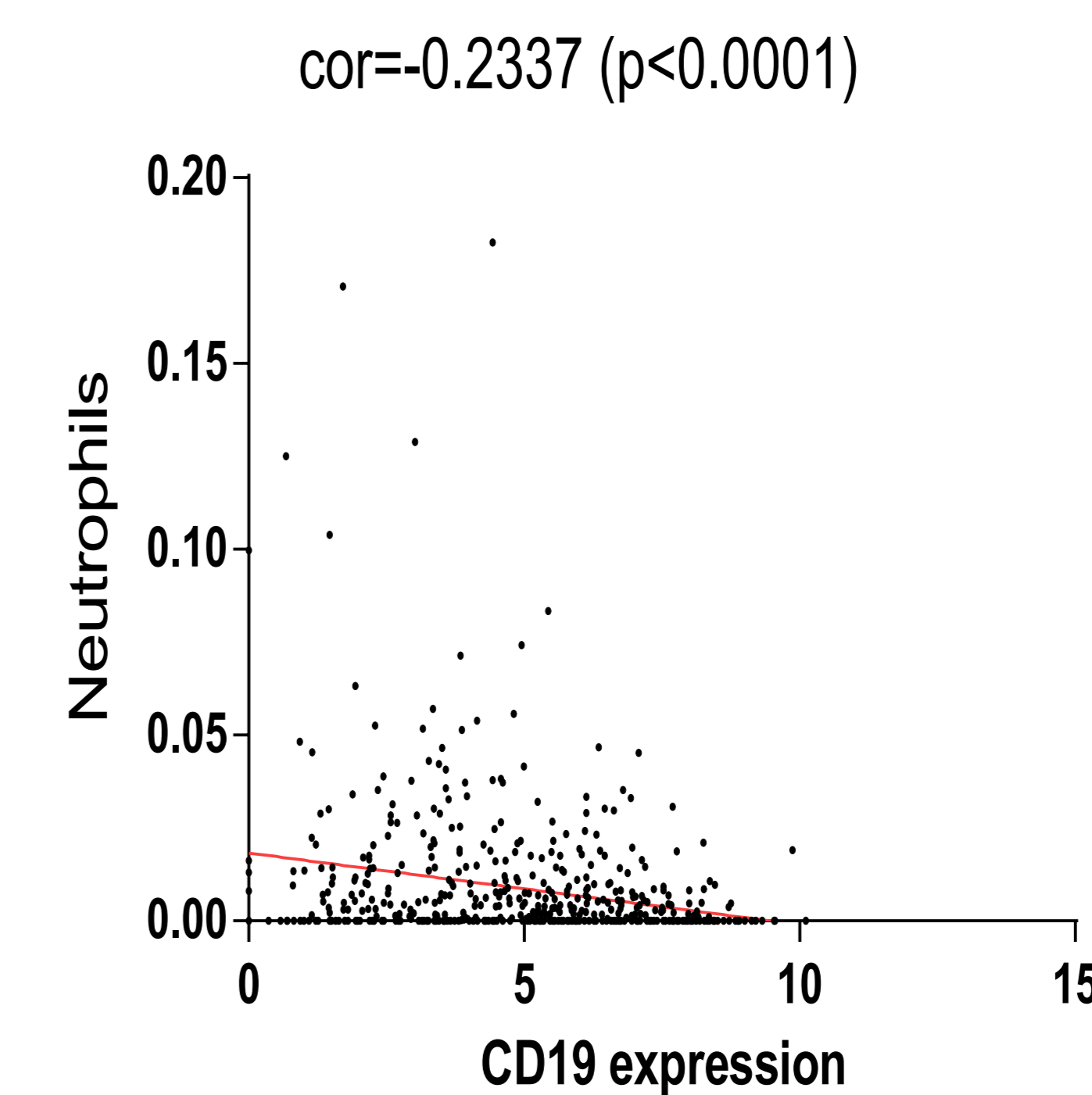

N

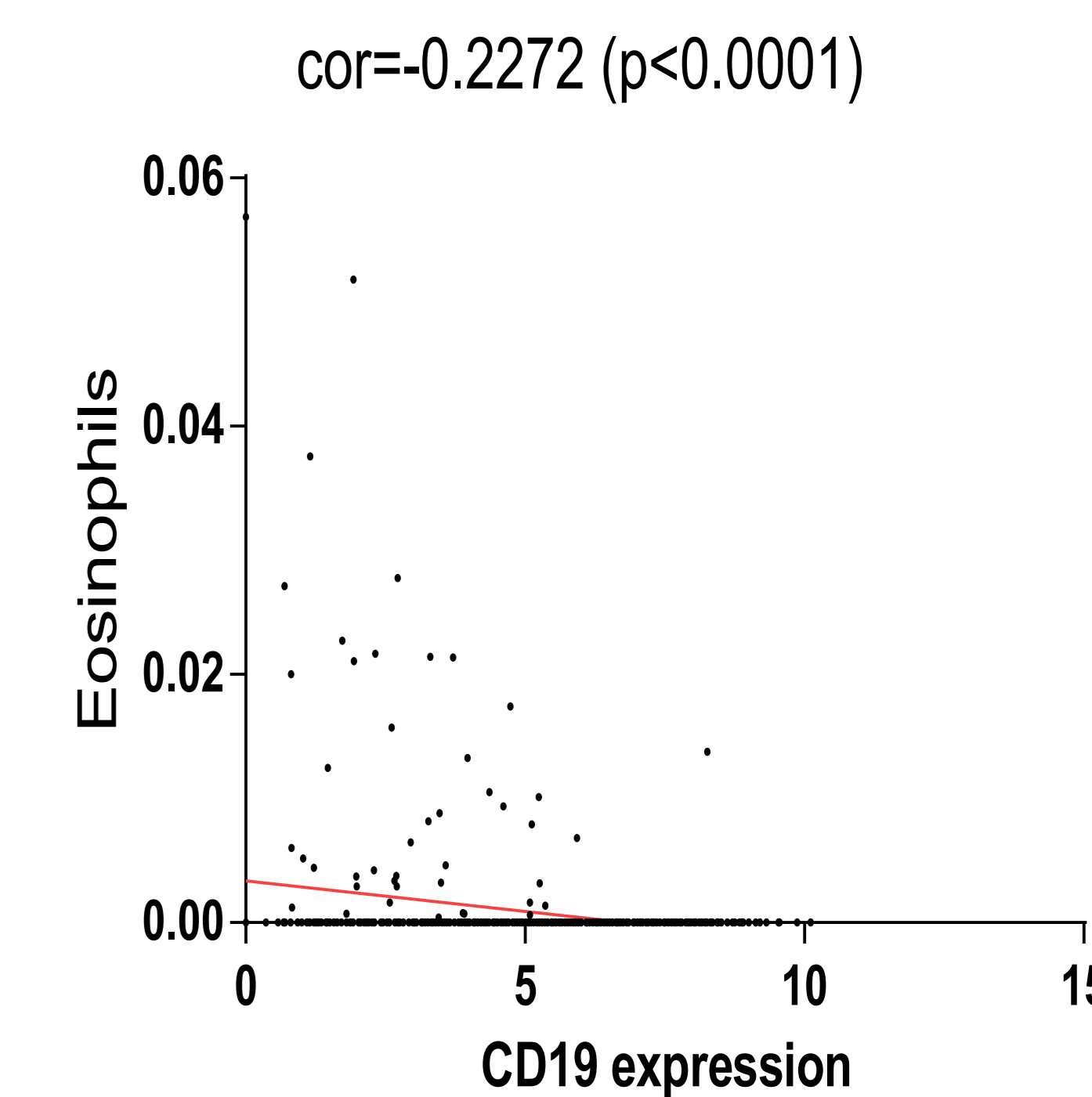

O

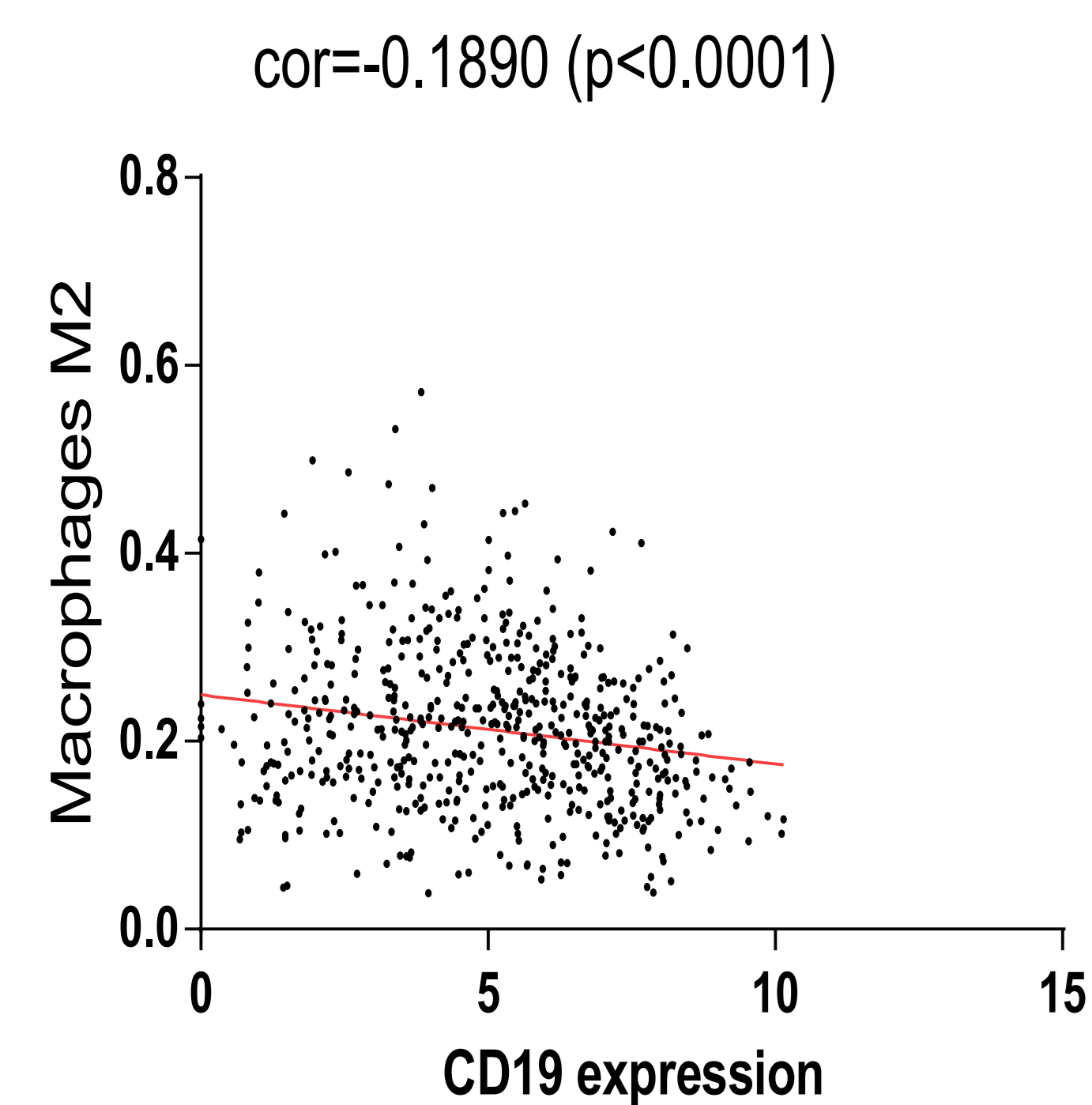

Supplement: Supplemental Information 3 — The red line in each plot was fifitted linear model indicating the proportion tropism of the immune cell along with CD19 expression, and Pearson coeffificient was used for the correlation test. [file peerj-09-10628-s003.pdf]
